# Supplementary material for: Exposure of western United States bird communities to predicted high severity fire
Source: Nat Commun. 2026 Jan 17;17:1775. doi: 10.1038/s41467-026-68480-7 (PMC12917125; doi:10.1038/s41467-026-68480-7)
Supplement: Supplementary file 1 — Supplementary Information [file 41467_2026_68480_MOESM1_ESM.pdf]

Supplementary Information: Exposure of western United States bird communities to predicted high severity fire

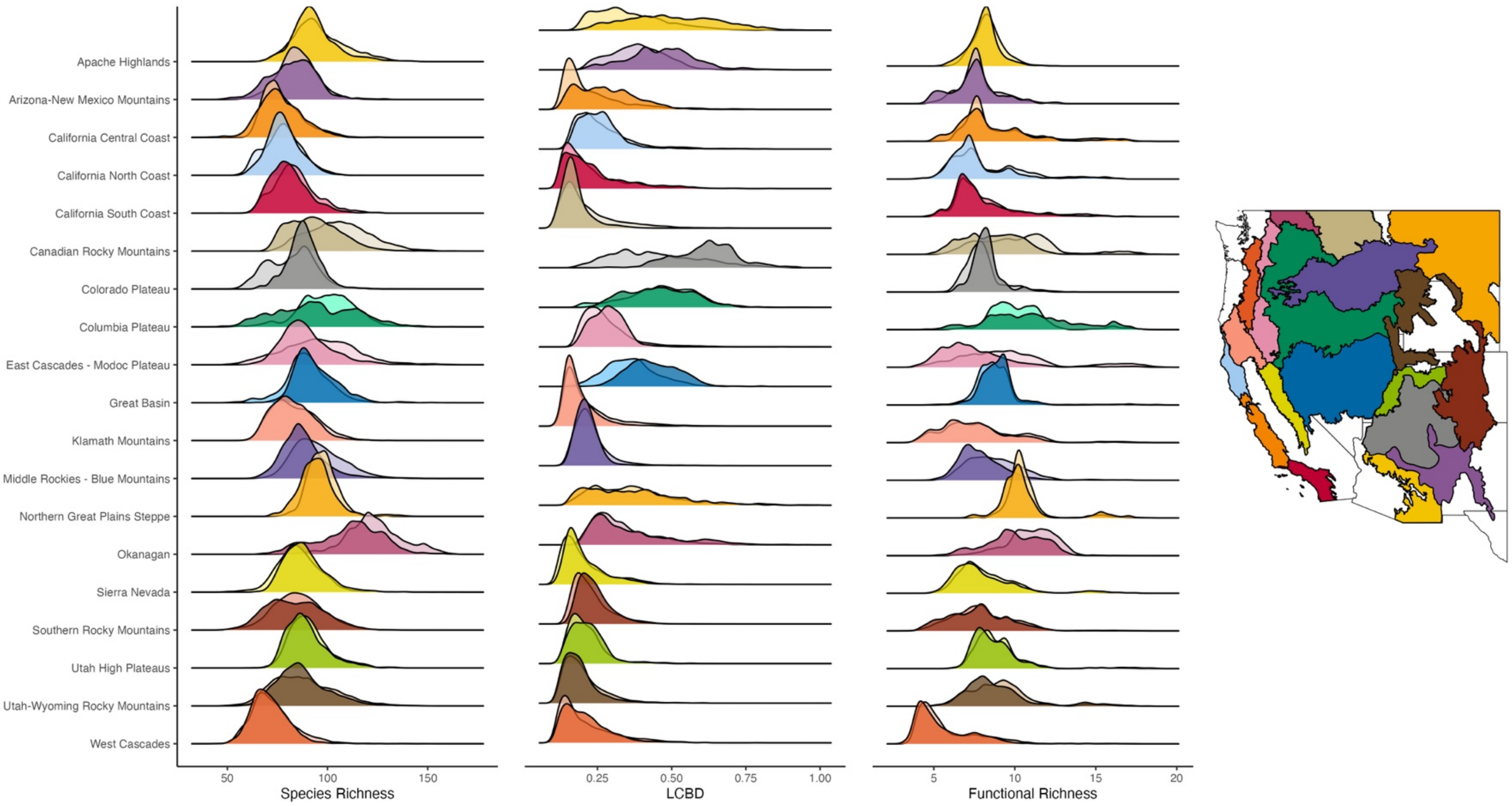

Figure S1. Bivariate density plots showing the distribution of biodiversity across low severity (lighter value colors) and high severity (darker value colors) areas. Each row corresponds to a single ecoregion, whose color also corresponds to the map on the far right for geographic context. Ecoregions generally fall into two groups, high congruence between high and low severity distributions (*Apache Highlands, Arizona-New Mexico Mountains, California South Coast, Great Basin, Klamath Mountains, Northern Great Plains Steppe, Sierra Nevada, Southern Rocky Mountains, Utah High Plateaus, Utah-Wyoming Rocky Mountains, West Cascades*) and differing in central tendency (*California Central Coast, California North Coast, Canadian Rocky Mountains, Colorado Plateau, Columbia Plateau, East Cascades, Middle Rockies – Blue Mountains, Okanagan*) categories, though the direction of the difference in central tendency sometimes differed between biodiversity metrics. Source data are provided as a Source Data file.

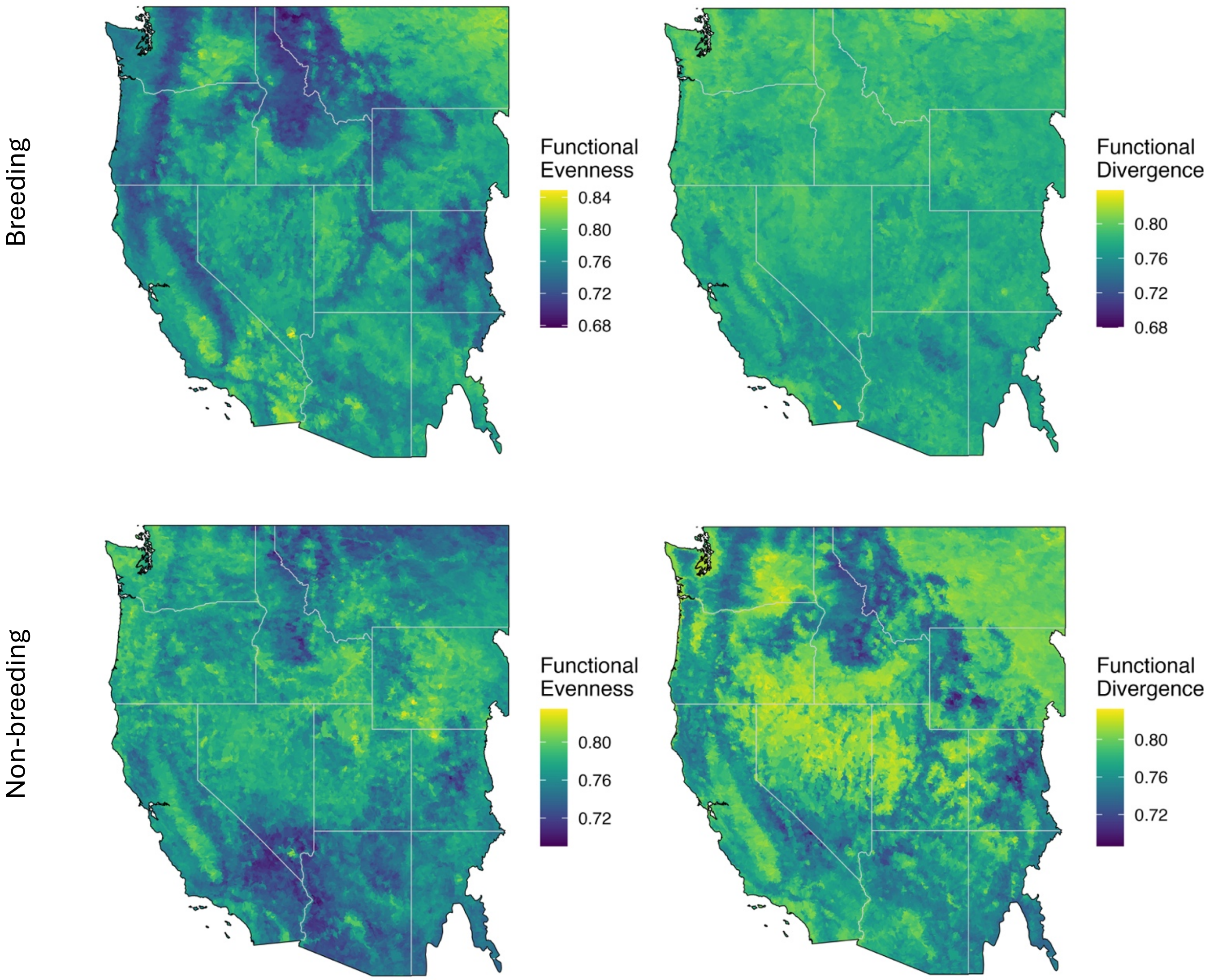

Figure S2: Functional evenness and divergence for breeding and non-breeding communities.

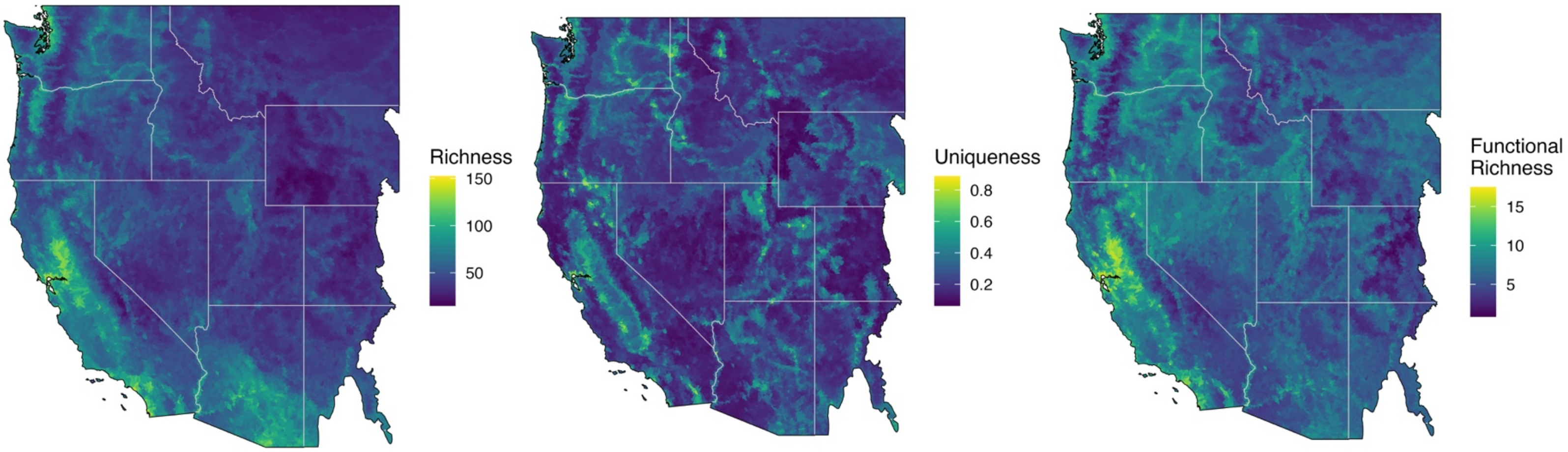

Figure S3: Species richness, community uniqueness, and functional richness for non-breeding communities.

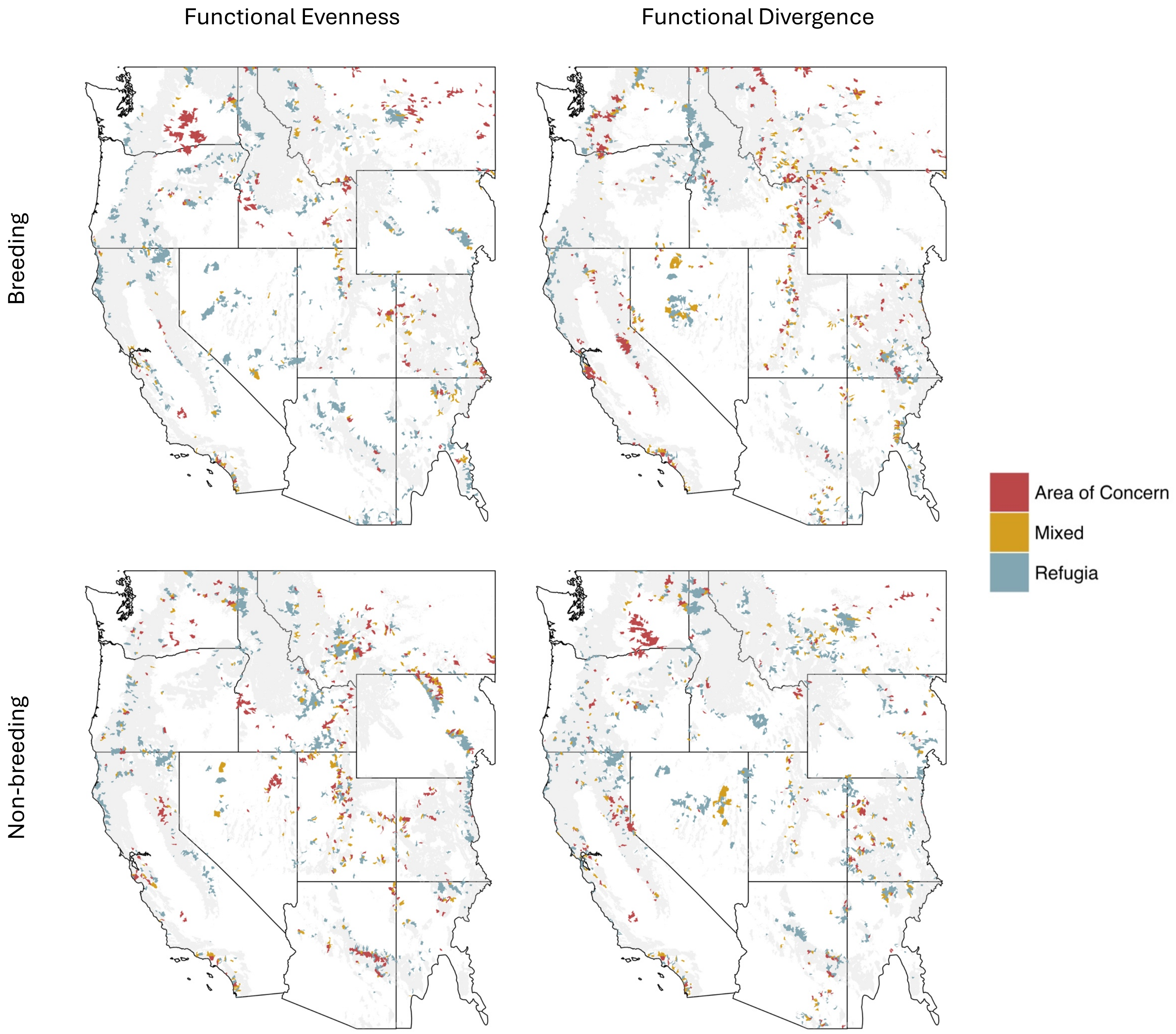

Figure S4: Hotspots for functional evenness and divergence for breeding and non-breeding communities.

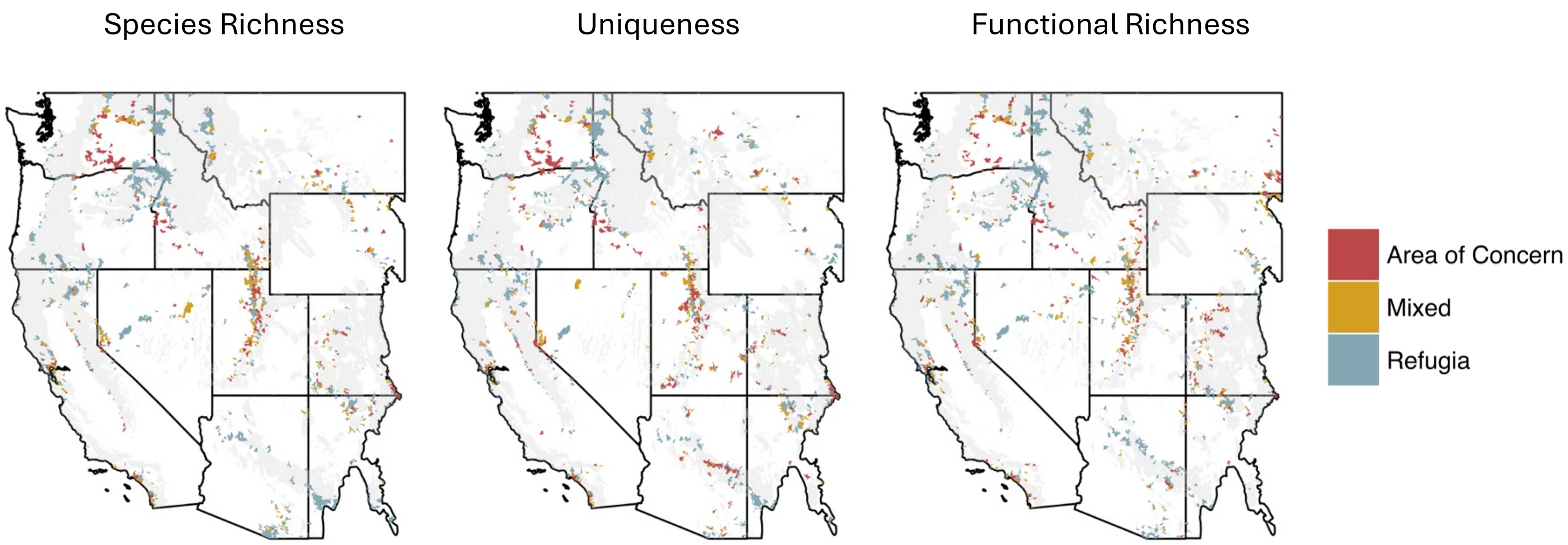

Figure S5: Hotspots for species richness, uniqueness, and functional richness of non-breeding communities.

Table S1: Statistics for beta regressions relating habitat traits to percent of forested population in high fire severity for breeding (n = 198) and nonbreeding (n = 126) communities, with the regression formula given by *percent high severity ~ habitat density + habitat*. Source data are provided as a Source Data file.

Breeding

	estimate	*std.error*	*statistic*	*p.value*
(Intercept)	-0.1	0.14	-0.76	0.45
Habitat.Density	-0.21	0.09	-2.41	0.02
HabitatGrassland	0.15	0.28	0.53	0.59
HabitatRiverine	0.46	0.43	1.08	0.28
HabitatRock	-0.07	0.3	-0.22	0.83
HabitatShrubland	0.07	0.12	0.63	0.53
HabitatWetland	0.45	0.32	1.41	0.16
HabitatWoodland	0.09	0.15	0.61	0.54
(phi)	24.19	3.37	7.17	0

Non-breeding

	estimate	*std.error*	*statistic*	*p.value*
(Intercept)	-0.32	0.2	-1.58	0.12
Habitat.Density	-0.14	0.13	-1.08	0.28
HabitatGrassland	0.37	0.35	1.08	0.28
HabitatRiverine	0.48	0.5	0.95	0.34
HabitatShrubland	0.22	0.18	1.21	0.23
HabitatWetland	0.3	0.51	0.59	0.55
HabitatWoodland	0.11	0.2	0.58	0.56
(phi)	19.01	3.31	5.75	0

Table S2: Statistics for beta regressions relating lifestyle traits to percent of forested population in high fire severity for breeding (n = 198) and nonbreeding (n = 126) communities, with the regression formula given by *percent in high severity ~ migration + trophic niche + primary lifestyle.* Source data are provided as a Source Data file.

Breeding

	estimate	*std.error*	*statistic*	*p.value*
(Intercept)	-0.41	0.46	-0.9	0.37
Migration	0.03	0.05	0.72	0.47
Trophic.NicheGranivore	-0.05	0.42	-0.12	0.91
Trophic.NicheHerbivore terrestrial	-0.11	0.46	-0.24	0.81
Trophic.NicheInvertivore	-0.01	0.42	-0.02	0.98
Trophic.NicheNectarivore	-0.05	0.5	-0.09	0.93
Trophic.NicheOmnivore	-0.1	0.41	-0.23	0.82
Trophic.NicheScavenger	-0.36	0.61	-0.59	0.56
Trophic.NicheVertivore	0.09	0.47	0.2	0.84
Primary.LifestyleAquatic	0.02	0.54	0.04	0.97
Primary.LifestyleGeneralist	0.1	0.23	0.43	0.67
Primary.LifestyleInsessorial	-0.06	0.21	-0.27	0.79
Primary.LifestyleTerrestrial	-0.3	0.26	-1.17	0.24
(phi)	25.53	3.56	7.17	0

Table S3: Statistics for beta regressions relating morphology traits to percent of forested population in high fire severity for breeding communities (n = 198), with the regression formula given by *percent in high severity ~ beak length + beak depth + tarus length + kipps distance + secondary 1 + hand wing index + tail length + log(mass)*. There was insufficient variation to fit lifestyle models for nonbreeding communities. Source data are provided as a Source Data file.

Breeding

	estimate	*std.error*	*statistic*	*p.value*
(Intercept)	-0.39	0.26	-1.51	0.13
Beak.Length_Culmen	0	0.01	-0.36	0.72
Beak.Depth	-0.04	0.02	-2.25	0.02
Tarsus.Length	0.01	0.01	0.84	0.4
Kipps.Distance	0	0	-0.38	0.7
Secondary1	0	0	0.85	0.4
Hand.Wing.Index	0	0.01	0.33	0.74
Tail.Length	0	0	-1.14	0.25
log(Mass)	-0.01	0.06	-0.12	0.91
(phi)	24.81	3.46	7.17	0

Non-breeding

	estimate	*std.error*	*statistic*	*p.value*
(Intercept)	-0.25	0.37	-0.67	0.5
Beak.Length_Culmen	0.01	0.01	1.04	0.3
Beak.Depth	-0.07	0.02	-3.19	0
Tarsus.Length	-0.01	0.01	-0.73	0.47
Kipps.Distance	0	0.01	0.25	0.8
Secondary1	0	0.01	0.56	0.58
Hand.Wing.Index	0	0.01	-0.15	0.88
Tail.Length	0	0	-0.4	0.69
log(Mass)	0.01	0.07	0.17	0.86
(phi)	22.81	3.98	5.73	0

Table S4. Parameters used to for bionomial test of hotspot distribution across severity levels relative to landscape distribution. Tests were performed for each hotspot type and ecoregion.

	Ecoregions	Hotspot cells in High severity	Total hotspot cells	Probability of being in high severity (percent of ecoregion in high severity)
**Uniqueness Hotspots**	California North Coast	209	1532	0.21972235519387300
	Canadian Rocky Mountains	331	3197	0.5255015711868500
	East Cascades - Modoc Plateau	172	1089	0.35251626052813000
	Klamath Mountains	267	2246	0.33273112601188200
	Arizona-New Mexico Mountains	1131	2674	0.14437570793761400
	Middle Rockies - Blue Mountains	409	1488	0.39639658690802200
	Okanagan	978	1229	0.5868062976419460
	Sierra Nevada	494	1421	0.39570757880617000
	Southern Rocky Mountains	120	422	0.4321138904827630
	Utah High Plateaus	180	351	0.6171541966779360
	Utah-Wyoming Rocky Mountains	480	1148	0.5474245844449010
	West Cascades	764	2506	0.41382861346083100
	Northern Great Plains Steppe	1369	2436	0.5438160348042260
	California Central Coast	650	1247	0.4553539916707840
	California South Coast	477	621	0.6701010303090930
	Apache Highlands	2484	3700	0.3693067085483510
	Colorado Plateau	2063	5588	0.26106583072100300
	Columbia Plateau	1538	5528	0.30367328313940200
	Great Basin	1819	2993	0.3546637744034710
**Richness Hotspots**	California North Coast	318	1513	0.21972235519387300
	Canadian Rocky Mountains	681	4707	0.5255015711868500
	East Cascades - Modoc Plateau	323	2094	0.35251626052813000
	Klamath Mountains	619	2766	0.33273112601188200
	Arizona-New Mexico Mountains	556	3809	0.14437570793761400
	Middle Rockies - Blue Mountains	1316	6803	0.39639658690802200
	Okanagan	298	1179	0.5868062976419460
	Sierra Nevada	1072	2192	0.39570757880617000
	Southern Rocky Mountains	2256	4848	0.4321138904827630
	Utah High Plateaus	699	1259	0.6171541966779360
	Utah-Wyoming Rocky Mountains	1464	3459	0.5474245844449010
	West Cascades	697	2618	0.41382861346083100
	Northern Great Plains Steppe	364	699	0.5438160348042260
	California Central Coast	698	1572	0.4553539916707840

	Ecoregions	Hotspot cells in High severity	Total hotspot cells	Probability of being in high severity (percent of ecoregion in high severity)
Functional Richness Hotspots	California South Coast	421	808	0.6701010303090930
	Apache Highlands	494	2132	0.3693067085483510
	Colorado Plateau	1486	4208	0.26106583072100300
	Columbia Plateau	1046	3492	0.30367328313940200
	Great Basin	1059	2221	0.3546637744034710
	California North Coast	243	1567	0.21972235519387300
	Canadian Rocky Mountains	437	4680	0.5255015711868500
	East Cascades - Modoc Plateau	224	1948	0.35251626052813000
	Klamath Mountains	564	2602	0.33273112601188200
	Arizona-New Mexico Mountains	423	3000	0.14437570793761400
	Middle Rockies - Blue Mountains	928	4173	0.39639658690802200
	Okanagan	289	955	0.5868062976419460
	Sierra Nevada	989	2346	0.39570757880617000
	Southern Rocky Mountains	1177	3317	0.4321138904827630
	Utah High Plateaus	538	1023	0.6171541966779360
	Utah-Wyoming Rocky Mountains	2042	3479	0.5474245844449010
	West Cascades	874	2702	0.41382861346083100
	Northern Great Plains Steppe	409	612	0.5438160348042260
	California Central Coast	448	1401	0.4553539916707840
	California South Coast	472	860	0.6701010303090930
	Apache Highlands	299	1202	0.3693067085483510
	Colorado Plateau	573	1831	0.26106583072100300
	Columbia Plateau	777	1452	0.30367328313940200
	Great Basin	190	511	0.3546637744034710
